# ﻿*Kodamaeahongheensis* f.a., sp. nov., *Kodamaeaovata* f.a., sp. nov. and *Kodamaeayamadae* f.a., sp. nov., three new yeast species of *Kodamaea* (Saccharomycetales, Debaryomycetacae) from China

**DOI:** 10.3897/mycokeys.89.81119

**Published:** 2022-04-29

**Authors:** Chun-Yue Chai, Wan-Li Gao, Ying Li, Zhen-Li Yan, Feng-Li Hui

**Affiliations:** 1 School of Life Science and Agricultural Engineering, Nanyang Normal University, Nanyang 473061, China Nanyang Normal University Nanyang China; 2 Research Center of Henan Provincial Agricultural Biomass Resource Engineering and Technology, Nanyang 473061, China Research Center of Henan Provincial Agricultural Biomass Resource Engineering and Technology Nanyang China; 3 State Key Laboratory of Motor Vehicle Biofuel Technology, Henan Tianguan Enterprise Group Co., Ltd., Nanyang 473000, China State Key Laboratory of Motor Vehicle Biofuel Technology Nanyang China

**Keywords:** Debaryomycetacae, 16 new combinations, Saccharomycetales, three new species, yeast taxonomy

## Abstract

*Kodamaea* includes a growing number of interesting yeasts of the family Debaryomycetacae that are widely distributed in temperate, subtropical and tropical regions of different continents. During recent yeast collections in Henan and Yunnan Province in China, several isolates of *Kodamaea* were obtained from rotting wood, all of which represent undescribed taxa. Based on morphological and phylogenetic analyses (ITS and LSU rDNA), three new species are proposed: *K.hongheensis* f.a., **sp. nov.**, *K.ovata* f.a., **sp. nov.** and *K.yamadae* f.a., **sp. nov.** In addition, sixteen *Candida* species, which are members of the *Kodamaea* clade based on phylogenetic analysis, are transferred to *Kodamaea* as new combinations. Our results indicate high species diversity of *Kodamaea* waiting to be discovered in rotting wood from tropical and subtropical China.

## ﻿Introduction

*Kodamaea* was proposed by Yamada et al. (1995a, b) to accommodate a single species, *K.ohmeri*, which was initially classified in the genus *Pichia*. Kurtzman (1998) did not accept this proposal because the entire genus *Pichia* was clearly polyphyletic and in need of careful revision. However, the discovery of four new ascosporogenous relatives of *K.ohmeri*, namely *K.anthophila*, *K.kakaduensis*, *K.laetipori* and *K.nitidulidarum*, added further justification to the recognition of *Kodamaea* as a separate genus (Lachance et al. 1999; Rosa et al. 1999; Suh and Blackwell 2005). Subsequently, four new anamorphic species of *Kodamaea*, *K.jinghongensis*, *K.meredithiae*, *K.neixiangensis* and *K.transpacifica* have been proposed as part of the genus based on their phylogenetic placement (Freitas et al. 2013; Sylvester et al. 2015; Gao et al. 2017). In addition, more than 16 species of *Candida* are recognized as members of the *Kodamaea* clade based on phylogenetic analysis of rDNA sequences (Hsieh et al. 2010; Lachance et al. 2011; Nakase et al. 2011; Daniel et al. 2014). The *Kodamaea* clade currently consists of nine species of the genus *Kodamaea* and 16 asexual species still assigned to the genus *Candida*, making it one of the growing numbers of interesting genera tentatively assigned to the family Metschnikowiaceae (Lachance and Kurtzman 2011; Nakase et al. 2011; Freitas et al. 2013; Daniel et al. 2014; Sylvester et al. 2015; Gao et al. 2017). On the basis of multigene phylogenetic analysis of nearly entire LSU rDNA, SSU rDNA, translation elongation factor-1a (*EF-1a*), two RNA polymerase II subunits gene (*RPB1* and *RPB2*) and whole genome, the *Kodamaea* clade has been found to be closely related to the *Aciculoconidium* clade and should be allocated to the family Debaryomycetacae (Kurtzman and Robnett 2013; Shen et al. 2018).

Species in *Kodamaea* are very common and inhabit a wide range of habitats, such as plant-related substrates, insects, insect frass, soil and seawater (Lachance and Kurtzman 2011; Lachance et al. 2011; Nakase et al. 2011; Freitas et al. 2013; Sylvester et al. 2015; Gao et al. 2017). Many of these species are associated with insects that occupy this ecological niche (Freitas et al. 2013; Suh and Blackwell 2005). They were isolated either directly from insects and insect frass or from common insect habitats, such as rotting wood, forest soil, mushrooms or flowers (Suh and Blackwell 2005; Hsieh et al. 2010; Lachance et al. 2011; Freitas et al. 2013; Gao et al. 2017). Significantly, *K.mesenterica* and *K.ohmeri* are also found in clinical specimens; it is thus possible that these species might act as emerging opportunistic pathogens (Lachance and Kurtzman 2011; Lachance et al. 2011).

Species of *Kodamaea* are widely distributed in temperate, subtropical and tropical regions of different continents, but most known species appear to exist in Asia, North America and South America (Suh and Blackwell 2005; Hsieh et al. 2010; Lachance et al. 2011; Nakase et al. 2011; Gao et al. 2017). The genus *Kodamaea* has been heavily studied in Asia, and 11 species of this clade were previously reported in Thailand, Japan and China. Among them, *K.loeiensis* is from Thailand (Nakase et al. 2011), while *K.fukazawae*, *K.fungicola* and *K.sagamina* are from Japan (Lachance et al. 2011). In China, *K.alishanica, K.hsintzibuensis*, *K.kaohsiungensis*, *K.lidongshanica* and *K.smagusa* are described from Taiwan Provinces (Hsieh et al. 2010), and *K.jinghongensis* and *K.neixiangensis* are from Henan and Yunnan Provinces (Gao et al. 2017). Our studies suggest the existence of other potentially new species of the genus in China. In this study, we collected rotting wood samples from Henan and Yunnan Provinces in China. After isolation and examination, three new species of *Kodamaea* were identified based on phenotypic characteristics and phylogenetic analysis, increasing the species diversity of *Kodamaea* in China.

## ﻿Materials and methods

### ﻿Sample collection and isolation

Samples of rotting wood were collected in the Xishuangbanna Primeval Forest Park (Yunnan Province, China) and the Baotianman Nature Reserve (Henan Province, China). The Xishuangbanna Primeval Forest Park (21°98'N, 100°88'E) is 1355 m above sea level (MASL), with a hot and humid climate. The average annual temperature is between 16 °C and 28 °C, and the average annual rainfall is above 1,100 mm. The Baotianman Nature Reserve (33°30'44"N, 111°55'47"E) is at 1830 (MASL), with a transitional climate from a northern subtropical zone to a warm temperate zone, average annual temperature of 14–16 °C, and average annual rainfall between 800 mm and 900 mm. Forty rotting wood samples were collected, twenty from each area, during July to August in 2016 and 2017. The samples were stored in sterile plastic bags and transported under refrigeration to the laboratory over a period of no more than 24 h. Yeast strains were isolated from rotting wood samples in accordance with the methods described by Gao et al. (2017) and Zheng et al. (2017). Each sample (1 g) was added to 20 ml sterile yeast extract-malt extract (YM) broth (0.3% yeast extract, 0.3% malt extract, 0.5% peptone, 1% glucose, pH 5.0 ± 0.2) supplemented with 0.025% sodium propionate and 200 mg/L chloramphenicol in a 150 ml Erlenmeyer flask and then cultured for 3–10 days on a rotary shaker. Subsequently, 0.1 ml aliquots of the enrichment culture and appropriate decimal dilutions were spread on YM agar plates and then incubated at 25 °C for 3–4 days. Different yeast colony morphotypes were then isolated by repeated plating on YM agar. All isolates were stored in Microbiology Lab of Nanyang Normal University (NYNU; Nanyang, China), and ex-type cultures of novel yeast were deposited in the fungal collection at Westerdijk Fungal Biodiversity Institute (CBS; Utrecht, The Netherlands) and China Centre of Industrial Culture Collection (CICC; Beijing, China).

### ﻿Morphological, physiological and biochemical studies

Morphological and physiological properties were determined according to Kurtzman et al. (2011). Carbon and nitrogen assimilation tests were performed using liquid media, and growth was observed for up to 4 weeks. Carbon fermentation was tested in a yeast extract peptone (YP) base media (1% yeast extract and 2% peptone, pH 5.0 ± 0.2), and Durham tubes were used to visualize carbon dioxide production. Growth at various temperatures (30 °C, 35 °C, 37 °C and 40 °C) was assessed by streaking cells onto yeast extract peptone glucose (YPD) agar (1% yeast extract, 2% peptone, 2% glucose, 2% agar, pH 5.0 ± 0.2) plates and incubating them for ~2 weeks. Formation of true hyphae and pseudohyphae was investigated using the Dalmau plate method on both cornmeal (CM) and 5% malt extract (ME) agar plates. Induction of the sexual stage was tested by incubating single or mixed cultures of the each of the two strains on YM agar, cornmeal (CM) agar, 5% malt extract (ME) agar, V8 agar, Gorodkowa agar, McClary’s acetate agar or yeast carbon base plus 0.01% ammonium sulphate (YCBAS) agar at 25 °C for 2 months (Lachance and Kurtzman 2011; Sylvester et al. 2015; Gao et al. 2017).

### ﻿DNA extraction, PCR amplification and nucleotide sequencing

Genomic DNA was extracted from the yeasts using the Ezup Column Yeast Genomic DNA Purification Kit according to the manufacturer’s protocol (Sangon Biotech, China). The nuclear rDNA ITS1-5.8S-ITS2 (ITS) region was amplified using the primer pair ITS1/ITS4 (White et al. 1990). The D1/D2 domain of LSU rDNA was amplified using the primer pair NL1/NL4 (Kurtzman and Robnett 1998). The following thermal profile was used to amplify the ITS and LSU rDNA regions: an initial denaturation step of 2 min at 95 °C, followed by 35 cycles of 30 s at 95 °C, 30 s at 51 °C, and 40 s at 72 °C, with a final extension of 10 min at 72 °C (Lv et al. 2020). PCR products were directly purified and sequenced by Sangon Biotech Inc. (Shanghai, China). We determined the identity and accuracy of the newly obtained sequences by comparing them to sequences in GenBank and assembled them using BioEdit (Hall 1999). Newly obtained sequences were then submitted to GenBank (https://www.ncbi.nlm.nih.gov/genbank/; Table 1).

**Table 1.** DNA sequences used in the molecular phylogenetic analysis. Entries in bold are newly generated in this study.

Species	Strain	Locality	Sample	ITS	D1/D2
*Kodamaeaarcana*	CBS 9883^T^	USA	Beetle	N/A	AY242347
*K.alishanica*	CBS 11429^T^	China	Soil	NR_159556	NG_063941
*K.derodonti*	CBS 9882^T^	USA	Beetle	NR_111388	AY242346
*K.fukazawae*	CBS 9137^T^	Japan	Mushroom	AB028033	AY313957
*K.fungicola*	CBS 9138^T^	Japan	Mushroom	AB028031	AY313958
*K.hsintzibuensis*	CBS 11427^T^	China	Soil	NR_160557	HQ999957
*K.kaohsiungensis*	CBS 11435^T^	China	Mushroom	NR_159557	HQ999958
*K.leandrae*	CBS 9735^T^	Brazil	Decaying fruit	NR_155222	AY449659
*K.lidongshanica*	CBS 11426^T^	China	Fruiting body	GU126451	HQ999959
*K.loeiensis*	CBS 11899^T^	Thailand	Insect frass	NR_155223	NG_073574
*K.mesenterica*	CBS 602^T^	Germany	Beer	NR_111297	U45720
*K.plutei*	CBS 9885^T^	USA	Beetle	NR_111389	AY520388
*K.restingae*	CBS 8493^T^	Brazil	Flowers Nitidulid beetles	NR_155225	AF059667
*K.sagamina*	CBS 9140^T^	Japan	Mushroom	AB028032	AY313959
*K.smagusa*	CBS 11430^T^	China	Mushroom	NR_111611	FJ873476
*K.suecica*	CBS 5724^T^	Sweden	Seawater	N/A	U45732
*K.anthophila*	CBS 8494^T^	Australia	Flowers, Nitidulid beetle	NR_155239	AF059668
*K.transpacifica*	CBS 12823^T^	Ecuador	Flowers	NR_173358	KF002564
*K.nitidulidarum*	CBS 8491^T^	Brazil	Flower Nitidulid beetle	NR_155241	AF059665
***K.ovata***	**NYNU 167144^T^**	**China**	**Rotting wood**	**OK381035**	**OK381037**
***K.ovata***	**NYNU 1685**	**China**	**Rotting wood**	**OM327522**	**OM327519**
*K.ohmeri*	CBS 5367^T^	USA	Cucumber brines	NR_121464	U45702
*K.jinghongensis*	CBS 14700^T^	China	Rotting wood	KY213814	KY213807
***K.hongheensis***	**NYNU 17423^T^**	**China**	**Rotting wood**	**MG255723**	**MG255704**
***K.hongheensis***	**NYNU 17409**	**China**	**Rotting wood**	**OM327517**	**OM327518**
*K.kakaduensis*	CBS 8611^T^	Australian	Flower	NR_155240	AF092279
*K.laetipori*	CBS 9884^T^	USA	Beetle	N/A	AY520398
*K.meredithiae*	CBS 13899^T^	USA	soil	OK050648	KM408122
*K.neixiangensis*	CBS 14699^T^	China	Rotting wood	KY213808	KY213820
***K.yamadae***	**NYNU 168114^T^**	**China**	**Rotting wood**	**OK381036**	**OK381034**
***K.yamadae***	**NYNU 16858**	**China**	**Rotting wood**	**OM327521**	**OM327516**
*Metschnikowialochheadii*	CBS 8807^T^	USA	flowers	NR_164507	NG_058341
*M.cubensis*	MUCL 45753^T^	Cuba	flowers	N/A	EU143316
*M.mataevar.maris*	CBS 13986^T^	Brazilian	flowers	N/A	KP241777
*M.cerradonensis*	CBS 10409^T^	Brazil	flowers nitidulid beetles	N/A	DQ641237
*M.continentalis*	CBS 8430^T^	Germany	flowers	N/A	DQ641238
*M.santaceciliae*	CBS 9149^T^	Costa Rica	nitidulid beetles	N/A	DQ641242
*M.borealis*	CBS 8431^T^	USA	beetles	N/A	DQ641243
*Aciculoconidiumaculeatum*	NRRL YB-4298^T^	USA	*Drosophilapinicola*	N/A	JQ689029
*Schizosaccharomycespombe*	NRRL Y-12796^T^	Jamaica, South Africa, Poland	Apple, Molass	KY105378	KY109602

Notes: Type strains are marked with T. N/A: sequences not available.

### ﻿Phylogenetic analyses

Species in the *Kodamaea* clade with high similarity to our new species were selected for phylogenetic analyses. *Schizosaccharomycespombe* NRRL Y-12796^T^ was used as an outgroup, based on Kuramae et al. (2006a). NCBI accession numbers of sequences used in the phylogenetic tree are listed in Table 1. Initial alignment of the combined ITS and partial LSU rDNA dataset was performed using the online version of MAFFT 6.0 (Katoh and Toh 2010) with manual evaluations and adjustments in BioEdit when necessary to obtain reliable and reasonable results (Hall 1999). The best-fit nucleotide substitution models for each gene were selected using jModelTest v2.1.7 (Darriba et al. 2012) according to the Akaike information criterion.

Neighbour-joining (NJ) and Maximum parsimony (MP) analyses were implemented for inferring the phylogenetic analyses by using MEGA software version 7.0 (Kumar et al. 2016). The NJ analysis was carried out using Kimura’s two parameter model (Kimura 1980) in the neighbour-joining method (Saitou and Nei 1987). Bootstrapping with 1,000 replicates was performed to determine branch support (Felsenstein 1985). The MP analysis was run using a heuristic search option of 1,000 search replicates with random-addition of sequences and tree bisection and reconnection (TBR) as the branch-swapping algorithm. NJ and MP bootstrap support values above 50% are shown as first and second positions above nodes, respectively.

## ﻿Results

### ﻿Phylogenetic analyses

**Figure 1.**
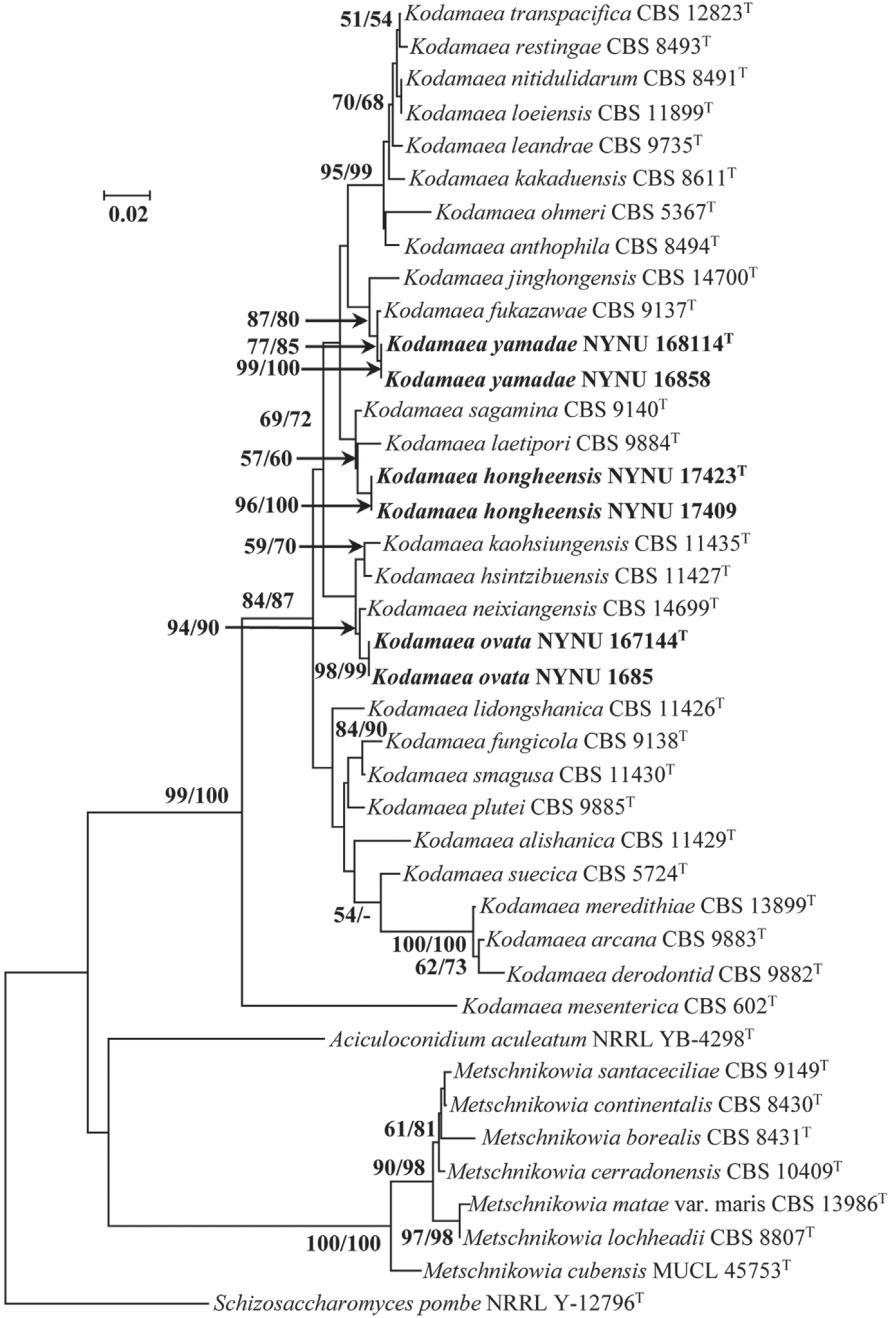
The NJ tree based on an analysis of a combined ITS and LSU rDNA dataset from the genus *Kodamaea* and related taxa from the family Debaryomycetacae. *Schizosaccharomycespombe* NRRL Y-12796^T^ was used as outgroup. Bootstrap support values (BS) for the neighbour-joining and maximum parsimony programs of above 50% are given at nodes based on 1000 replications, a dash (“-”) indicates a value < 50% (BS). Bar, 2% sequence difference. The strain number is indicated after the species name. The strains in this study are in bold. T, type strains.

The combined ITS and LSU rDNA sequences dataset was analysed to infer the interspecific relationships within the *Kodamaea* clade of the family Debaryomycetacae. The dataset consisted of 40 sequences including the outgroup, *Schizosaccharomycespombe* NRRL Y-12796^T^. A total of 896 characters including gaps (372 for ITS and 524 for LSU rDNA) were included in the phylogenetic analysis. Both NJ and MP analyses resulted in similar tree topologies, and only the NJ tree is shown in Fig. 1.

In the NJ phylogenetic tree (Fig. 1), the genus *Kodamaea* formed a monophyletic clade distant from its related taxa of the family Debaryomycetacae. The samples of the three new species of *Kodamaea*, *Kodamaeahongheensis*, *Kodamaeaovata* and *Kodamaeayamadae*, formed each a strongly supported sub-clade and were clearly distinct from other known species of *Kodamaea*. Two strains of *K.hongheensis* formed a unique lineage with *K.laetipori*, but with low support (NJ 57%, MP 60%). *K.ovata* clustered with *K.neixiangensis* with high support (NJ 94%, MP 90%), while *K.yamadae* clustered with *K.jinghongensis* and *K.fukazawae* with evident statistic support (NJ 87%, MP 80%).

### ﻿Taxonomy

#### ﻿ Kodamaea hongheensis

Taxon classificationFungiSaccharomycetalesDebaryomycetacae

C.Y. Chai & F.L. Hui sp. nov.

C777932A-7DFE-58E2-BC05-0A6124899482

842625

Fig. 2

##### Holotype.

China, Yunnan Province, Honghe Prefecture, Luxi County, in rotting wood in Jiuxi Mountain Forest Park, April 2017, K.F. Liu & Z.W. Xi (holotype NYNU 17423^T^, ex-holotype CICC 33265).

##### Etymology.

The specific epithet *hongheensis* refers to the geographic origin of the type strain, Honghe Prefecture, Yunnan.

##### Description.

**Figure 2.**
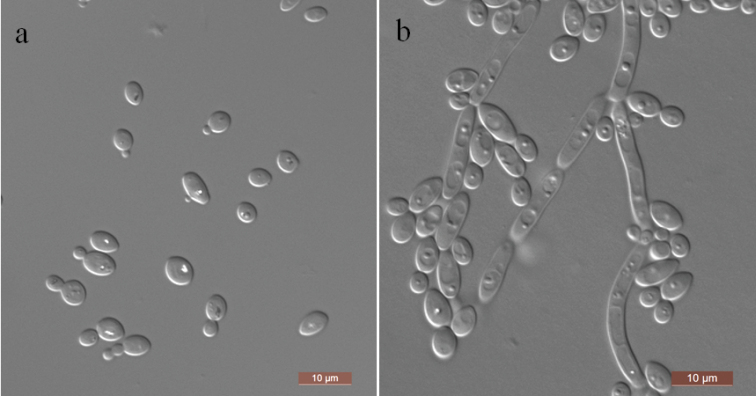
Morphology of *Kodamaeahongheensis* (NYNU 17423, holotype) **a** budding cells in YM broth after 3 d **b** simple pseudohyphae on CM agar after 14 d. Scale bars: 10 μm.

In YM broth, after 3 days at 25 °C, cells are ovoid to elongate (3–5 × 3–7 μm) and occur singly or in pairs (Fig. 2a). Sediment is formed after a month, but a pellicle is not observed. On YM agar, after 3 days at 25 °C, colonies are white to cream-colored, butyrous and smooth with entire margins. In Dalmau plate culture on CM agar, pseudohyphae are formed but not true mycelia (Fig. 2b). Asci or signs of conjugation are not seen on sporulation media. Glucose, maltose and trehalose are fermented but not galactose, sucrose, melibiose, lactose, cellobiose, melezitose, raffinose, inulin, or xylose. Glucose, glucosamine, d-xylose, sucrose, maltose, trehalose, methyl α-d-glucoside, cellobiose, salicin, melezitose, glycerol, ribitol, d-glucitol, d-mannitol, d-glucono-1, 5-lactone, 2-keto-d-gluconate, succinate, citrate and ethanol are assimilated as sole carbon sources. Galactose, l-sorbose, d-ribose, d-arabinose, l-arabinose, l-rhamnose, melibiose, lactose, raffinose, inulin, erythritol, xylitol, galactitol, *myo*-inositol, d-gluconate, d-glucuronate, dl-lactate and methanol are not assimilated. l-lysine, glucosamine and d-tryptophan are assimilated as sole nitrogen sources. Nitrate, nitrite, ethylamine, cadaverine, creatine, creatinine and imidazole are not assimilated. Growth is observed at 35 °C but not at 37 °C. Growth in the presence of 0.01% cycloheximide is positive, but growth in the presence of 10% NaCl plus 5% glucose and 1% acetic acid is negative. Starch-like compounds are not produced. Urease activity and diazonium blue B reactions are negative.

##### Additional isolate examined.

China, Yunnan Province, Honghe Prefecture, Luxi County, in rotting wood in Jiuxi Mountain Forest Park, April 2017, K.F. Liu & Z.W. Xi (NYNU 17409).

##### Notes.

Two strains NYNU 17409 and NYNU 17423, both representing *K.hongheensis*, were grouped in an independent lineage and are related to *K.laetipori*. *K.hongheensis* differed from its closest relative *K.laetipori* by 2.9% substitutions in the LSU rDNA sequence. However, the ITS sequence of *K.hongheensis* could not be successfully aligned with the type strain of *K.laetipori* because its ITS sequences are not currently available from either the NCBI GenBank database or the CBS database. Physiologically, *K.hongheensis* can be differentiated from *K.laetipori* (Lachance and Kurtzman 2011) based on growth in l-sorbose, d-arabinose, d-gluconate, and dl-lactate, which are positive for *K.laetipori* and negative for the new species. Moreover, *K.laetipori* grows in the presence of 0.1% cycloheximide and 10% NaCl plus 5% glucose, but *K.hongheensis* does not.

#### ﻿ Kodamaea ovata

Taxon classificationFungiSaccharomycetalesDebaryomycetacae

C.Y. Chai & F.L. Hui sp. nov.

A717199B-8187-5328-927A-89B909182552

842623

Fig. 3

##### Holotype.

China, Henan Province, Nanyang City, the Baotianman Nature Reserve, in rotting wood under a mixed forest, July 2016, K.F. Liu & Z.W. Xi (holotype NYNU 167144^T^, ex-holotype CBS 14702).

##### Etymology.

The specific epithet *ovata* refers to the ovoid cell morphology of the type strain.

##### Description.

**Figure 3.**
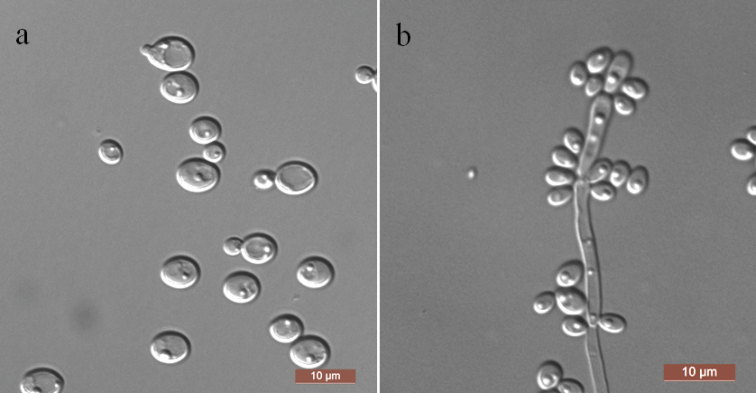
Morphology of *Kodamaeaovata* (NYNU 167144, holotype) **a** budding cells in YM broth after 3 d **b** pseudohyphae on CM agar after 14 d. Scale bars: 10 μm.

In YM broth, after 3 days at 25 °C, cells are ovoid (2–4 × 3–5 μm) and occur singly or in pairs (Fig. 3a). Sediment is formed after a month, but a pellicle is not observed. On YM agar, after 3 days at 25 °C, colonies are white to cream-colored, butyrous and smooth with entire margins. In Dalmau plate culture on CM agar, a rudimentary pseudomycelium is formed (Fig. 3b). Asci or signs of conjugation are not seen on sporulation media. Glucose, galactose, maltose and trehalose are fermented but sucrose, melibiose, lactose, cellobiose, melezitose, raffinose, inulin and xylose are not. Glucose, galactose, glucosamine, d-xylose, d-arabinose, l-arabinose, sucrose, maltose, trehalose, methyl α-d-glucoside, cellobiose, salicin, arbutin, melezitose, inulin, glycerol, ribitol, xylitol, d-glucitol, d-mannitol, galactitol, d-glucono-1, 5-lactone, 2-keto-d-gluconate, dl-lactate, succinate, citrate and ethanol are assimilated as sole carbon sources. l-sorbose, d-ribose, l-rhamnose, melibiose, lactose, raffinose, erythritol, *myo*-inositol, d-gluconate, d-glucuronate and methanol are not assimilated. Ethylamine, l-lysine and creatine are assimilated as sole nitrogen sources. Nitrate, nitrite, cadaverine, creatinine, glucosamine, imidazole and d-tryptophan are not assimilated. Growth is observed at 42 °C but not at 45 °C. Growth in the presence of 0.1% cycloheximide and 16% NaCl plus 5% glucose is positive, but growth in the presence of 1% acetic acid is negative. Starch-like compounds are not produced. Urease activity and diazonium blue B reactions are negative.

##### Additional isolate examined.

China, Henan Province, Nanyang City, the Baotianman Nature Reserve, in rotting wood under a mixed forest, July 2016, K.F. Liu & Z.W. Xi (NYNU 1685).

##### Notes.

Two strains NYNU 1685 and NYNU 167144 representing *K.ovata* grouped in a well-supported clade and appear to be most closely related to *K.neixiangensis* (Gao et al. 2017). The nucleotide differences between the new species and the close relative *K.neixiangensis* are 1% substitutions in the LSU rDNA sequence and 4.8% substitutions in the ITS region, respectively. Physiologically, *K.ovata* can be differentiated from *K.neixiangensis* based on growth in l-arabinose, d-arabinose, dl-lactate and 16% NaCl plus 5% glucose, all of which were positive for *K.ovata* and negative for *K.neixiangensis*. Additionally, the new species ferments galactose and maltose and grows at 35 °C, but *K.neixiangensis* does not have these characteristics.

#### ﻿ Kodamaea yamadae

Taxon classificationFungiSaccharomycetalesDebaryomycetacae

C.Y. Chai & F.L. Hui sp. nov.

97F810A2-3E72-510A-AD92-76803C758104

842626

Fig. 4

##### Holotype.

China, Henan Province, Nanyang City, the Baotianman Nature Reserve, in rotting wood under a mixed forest, August 2016, K.F. Liu & Z.W. Xi (holotype NYNU 168114^T^, ex-holotype CBS 14703).

##### Etymology.

The specific epithet *yamadae* is used in honour of Y. Yamada for his proposal of the genus *Kodamaea*.

##### Description.

**Figure 4.**
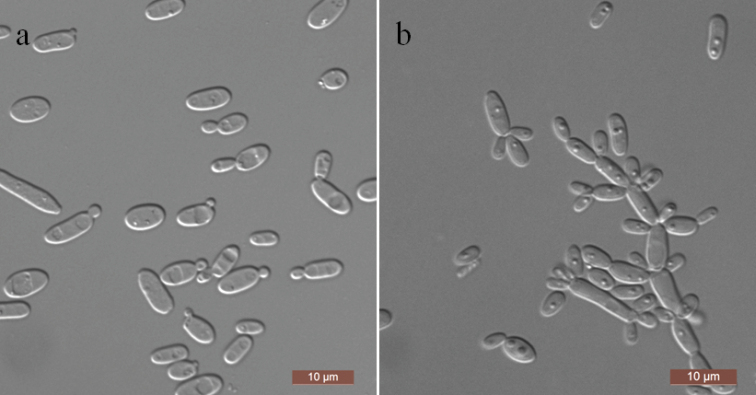
Morphology of *Kodamaeayamadae* (NYNU 168114, holotype) **a** budding cells in YM broth after 3 d **b** pseudohyphae on CM agar after 14 d. Scale bars: 10 μm.

In YM broth, after three days at 25 °C, cells are ellipsoidal to elongate (2–3 × 4.5–10 μm) and occur singly or in pairs (Fig. 4a). Sediment is formed after a month, but a pellicle is not observed. On YM agar, after 3 days at 25 °C, colonies are white, convex, sometimes fringed, glabrous or membranous, smooth or rugose and butyrous to tough due to filamentous growth. On Dalmau plate culture on CM agar, a rudimentary pseudomycelium is formed (Fig. 4b). Asci or signs of conjugation are not seen on sporulation media. Glucose, maltose, sucrose, trehalose and cellobiose are fermented but not galactose, melibiose, lactose, melezitose, raffinose, inulin or xylose. Glucose, galactose, glucosamine, d-ribose, d-xylose, sucrose, maltose, trehalose, methyl α-d-glucoside, cellobiose, salicin, inulin, glycerol, erythritol, ribitol, d-glucitol, d-mannitol, d-glucono-1, 5-lactone, 2-keto-d-gluconate, succinate, citrate and ethanol are assimilated as sole carbon sources. l-sorbose, d-arabinose, l-arabinose, l-rhamnose, arbutin, melibiose, lactose, raffinose, melezitose, xylitol, galactitol, *myo*-inositol, 5-keto-d-gluconate, d-gluconate, d-glucuronate, dl-lactate and methanol are not assimilated. Ethylamine, l-lysine, creatine, glucosamine and d-tryptophan are assimilated as sole nitrogen sources. Nitrate, nitrite, cadaverine, creatinine and imidazole are not assimilated. Growth is observed at 30 °C but not at 35 °C. Growth in the presence of 0.1% cycloheximide is positive, but growth in the presence of 10% NaCl plus 5% glucose and 1% acetic acid is negative. Starch-like compounds are not produced. Urease activity and diazonium blue B reactions are negative.

##### Additional isolates examined.

China, Henan Province, Nanyang City, the Baotianman Nature Reserve, in rotting wood under an oak forest, August 2016, K.F. Liu & Z.W. Xi (NYNU 16858).

##### Notes.

Two strains NYNU 16858 and NYNU 168114, representing *K.yamadae* clustered in a well-supported clade that is closely related to *K.jinghongensis* (Gao et al. 2017) and *K.fukazawae* (Nakase et al. 1999). The nucleotide differences between *K.yamadae* and *K.jinghongensis* were 2.8% substitutions in the LSU rDNA sequences and 3.9% substitutions in the ITS region. Similarly, *K.yamadae* and *K.fukazawae* showed differences of 2.6% substitutions in the LSU rDNA sequences and 4.7% substitutions in the ITS region. Physiologically, the novel species differed from *K.jinghongensis* by its ability to ferment cellobiose and its inability to assimilate arbutin. Unlike *K.fukazawae*, the novel species was able to assimilate d-galactose, l-sorbose, inulin, d-arabinose, l-arabinose, l-rhamnose, and methanol, and was not able to grow in the presence of 0.1% cycloheximide. In all cases, identification by sequencing was the best approach.

### ﻿Sixteen new combinations

In addition to the previously described taxa, we propose sixteen new combinations in the genus *Kodamaea* by including clade members that previously were described as species of the polyphyletic asexual genus *Candida* based on the combined ITS and LSU rDNA sequences from type strains of the genus *Kodamaea* and related taxa of the family Debaryomycetacae.

#### ﻿ Kodamaea alishanica

Taxon classificationFungiSaccharomycetalesDebaryomycetacae

(C.W. Hsieh) C.Y. Chai & F.L. Hui comb. nov.

DAB749F7-5472-5B51-838D-804DC48DE0A9

843566

##### Basionym.

*Candidaalishanica* C.W. Hsieh, FEMS Yeast Research 10 (7): 948 (2010).

#### ﻿ Kodamaea arcana

Taxon classificationFungiSaccharomycetalesDebaryomycetacae

(S.-O. Suh & M. Blackw) C.Y. Chai & F.L. Hui comb. nov.

6730D984-42E4-5E1E-BBE3-0DB352ADD61E

843565

##### Basionym.

*Candidaarcana* S.-O. Suh & M. Blackw, Mycologia 97 (1): 172 (2005).

#### ﻿ Kodamaea derodonti

Taxon classificationFungiSaccharomycetalesDebaryomycetacae

(S.-O. Suh & M. Blackw) C.Y. Chai & F.L. Hui comb. nov.

FD18B33A-A651-54B2-BAC3-AA7F68841883

843567

##### Basionym.

*Candidaderodonti* S.-O. Suh & M. Blackw, Mycologia 97 (1): 172 (2005).

#### ﻿ Kodamaea fukazawae

Taxon classificationFungiSaccharomycetalesDebaryomycetacae

(Nakase, M. Suzuki, Sugita, S.O. Suh & Komag) C.Y. Chai & F.L. Hui comb. nov.

5F59437E-7751-54A0-B4E6-237E95DCFA61

843579

##### Basionym.

*Candidafukazawae* Nakase, M. Suzuki, Sugita, S.O. Suh & Komag, Mycoscience 40 (6): 473 (1999).

#### ﻿ Kodamaea fungicola

Taxon classificationFungiSaccharomycetalesDebaryomycetacae

(Nakase, M. Suzuki, Sugita, S.O. Suh & Komag) C.Y. Chai & F.L. Hui comb. nov.

EE55CBC3-C2FF-5B59-AF8D-10FA23A1DEC1

843568

##### Basionym.

*Candidafungicola* Nakase, M. Suzuki, Sugita, S.O. Suh & Komag, Mycoscience 40 (6): 470 (1999).

#### ﻿ Kodamaea hsintzibuensis

Taxon classificationFungiSaccharomycetalesDebaryomycetacae

(C.W. Hsieh) C.Y. Chai & F.L. Hui comb. nov.

33020801-09D9-5F04-9BE8-E0D49BB17A0E

843569

##### Basionym.

*Candidahsintzibuensis* C.W. Hsieh, FEMS Yeast Research 10 (7): 948 (2010).

#### ﻿ Kodamaea kaohsiungensis

Taxon classificationFungiSaccharomycetalesDebaryomycetacae

(C.W. Hsieh) C.Y. Chai & F.L. Hui comb. nov.

AA98F30E-1379-5100-A50B-C175EFA171D3

843570

##### Basionym.

*Candidakaohsiungensis* C.W. Hsieh, FEMS Yeast Research 10 (7): 948 (2010).

#### ﻿ Kodamaea leandrae

Taxon classificationFungiSaccharomycetalesDebaryomycetacae

(Ruivo, Pagnocca, Lachance & Rosa) C.Y. Chai & F.L. Hui comb. nov.

508BCF0D-78DE-5897-933A-DB15CFF244D9

842628

##### Basionym.

*Candidaleandrae* Ruivo, Pagnocca, Lachance & Rosa, International Journal of Systematic and Evolutionary Microbiology 54(6): 62407 (2004).

#### ﻿ Kodamaea lidongshanica

Taxon classificationFungiSaccharomycetalesDebaryomycetacae

(C.W. Hsieh) C.Y. Chai & F.L. Hui comb. nov.

9F88066B-F09B-586D-8801-50730878DC29

843571

##### Basionym.

*Candidalidongshanica* C.W. Hsieh, FEMS Yeast Research 10 (7): 948 (2010).

#### ﻿ Kodamaea loeiensis

Taxon classificationFungiSaccharomycetalesDebaryomycetacae

(Nakase, Jindamorakot, Am-In, Ninomiya & Kawasaki) C.Y. Chai & F.L. Hui comb. nov.

35B8E584-4B9B-516A-B5B1-3C140730C887

842627

##### Basionym.

*Candidaloeiensis* Nakase, Jindamorakot, Am-In, Ninomiya & Kawasaki, Journal of General and Applied Microbiology 57(6): 2011(388).

#### ﻿ Kodamaea mesenterica

Taxon classificationFungiSaccharomycetalesDebaryomycetacae

((A. Geiger) Diddens & Lodder) C.Y. Chai & F.L. Hui comb. nov.

6F3B96DF-DAC1-5832-971C-6131D83C2893

843572

##### Basionym.

*Candidamesenterica* (A. Geiger) Diddens & Lodder, Die anaskosporogenen Hefen, II Hälfte: 196 (1942).

#### ﻿ Kodamaea plutei

Taxon classificationFungiSaccharomycetalesDebaryomycetacae

(S.-O. Suh & M. Blackw) C.Y. Chai & F.L. Hui comb. nov.

F5A30450-4EA2-584E-BDB2-D3139F0602D9

843573

##### Basionym.

*Candidaplutei* S.-O. Suh & M. Blackw, Mycologia 97 (1): 173 (2005)

#### ﻿ Kodamaea restingae

Taxon classificationFungiSaccharomycetalesDebaryomycetacae

(Rosa, Lachance, Starmer, Barker, Bowles & Schlag-Edler) C.Y. Chai & F.L. Hui comb. nov.

E2A848BC-BF5C-5B41-B210-F899AF75560E

842629

##### Basionym.

*Candidarestingae* Rosa, Lachance, Starmer, Barker, Bowles & Schlag-Edler, International Journal of Systematic Bacteriology 49(1):313 (1999).

#### ﻿ Kodamaea sagamina

Taxon classificationFungiSaccharomycetalesDebaryomycetacae

(Nakase, M. Suzuki, Sugita, S.O. Suh & Komag) C.Y. Chai & F.L. Hui comb. nov.

FCF96A1E-1FAC-57BD-B3E1-713DA563959B

843575

##### Basionym.

*Candidasagamina* Nakase, M. Suzuki, Sugita, S.O. Suh & Komag, Mycoscience 40 (6): 471 (1999).

#### ﻿ Kodamaea smagusa

Taxon classificationFungiSaccharomycetalesDebaryomycetacae

(C.W. Hsieh) C.Y. Chai & F.L. Hui comb. nov.

B73853E5-2C6B-59AF-ACB2-5E7B688E96DC

843576

##### Basionym.

*Candidasmagusa* C.W. Hsieh, FEMS Yeast Research 10 (7): 948 (2010).

#### ﻿ Kodamaea suecica

Taxon classificationFungiSaccharomycetalesDebaryomycetacae

(Rodr. Mir. & Norkrans) C.Y. Chai & F.L. Hui comb. nov.

92CE5713-990D-5004-B2A6-AD67A2C15F6E

843577

##### Basionym.

*Candidasuecica* Rodr. Mir. & Norkrans, Antonie van Leeuwenhoek 34: 115 (1968).

## ﻿Discussion

In this study, three new species of *Kodamaea*, namely *Kodamaeahongheensis* f.a., sp. nov., *Kodamaeaovata* f.a., sp. nov. and *Kodamaeayamadae* f.a., sp. nov., from Henan and Yunnan Province in China are described and compared with similar species based on morphological and molecular data. A more comprehensive phylogenetic placement of the genus *Kodamaea* based on the combined ITS and LSU rDNA sequences is provided, including almost all representatives from GenBank database and newly generated sequences. This study provides some ideas on the species delimitation within *Kodamaea* based on morphological and phylogenetic placement evidence.

The phylogenetic relationships in *Kodamaea* have been unclear, mainly due to lacking a multigene phylogeny (Daniel et al. 2014). In this article, we used all currently known species and the new species to revise this genus, based on a phylogenetic analysis of the combined ITS and LSU rDNA sequences. As shown in Fig. 1, the genus *Kodamaea* formed a monophyletic clade with well support (NJ 99%, MP 100%). This result is similar to the results of previous phylogenetic analyses based on the D1/D2 domain of LSU rDNA sequences (Freitas et al. 2013; Gao et al. 2017). According to the nomenclature of “one fungus, one name”, sixteen asexual *Candida* species, which are members of the *Kodamaea* clade based on phylogenetic analysis, are transferred to *Kodamaea* as *K.alishanica* comb. nov., *K.arcana* comb. nov., *K.derodonti* comb. nov., *K.fukazawae* comb. nov., *K.fungicola* comb. nov., *K.hsintzibuensis* comb. nov., *K.kaohsiungensis* comb. nov., *K.leandrae* comb. nov., *K.lidongshanica* comb. nov., *K.loeiensis* comb. nov., *K.mesenterica* comb. nov., *K.plutei* comb. nov., *K.restingae* comb. nov., *K.sagamina* comb. nov., *K.smagusa* comb. nov. and *K.suecica* comb. nov.

In recent years, many new yeast species have been identified from rotting wood in China (Gao et al. 2017; Zheng et al. 2017; Lv et al. 2020). However, there is still a large number of undescribed yeast taxa in China. This study indicates that there are at least five species of *Kodamaea* isolated from rotting wood in China, including two species known previously to occur in China (*K.jinghongensis* and *K.neixiangensis*), and three novel species (*K.hongheensis*, *K.ovata* and *K.yamadae*). In China, there are still some species that need to be discovered, such as those listed under GenBank accessions KM598654 and HQ623482. Our study indicates that there is high species diversity of *Kodamaea* waiting to be discovered in rotting wood in tropical and subtropical China and nearby areas as with other genera (Lv et al. 2020).

## Supplementary Material

XML Treatment for Kodamaea hongheensis

XML Treatment for Kodamaea ovata

XML Treatment for Kodamaea yamadae

XML Treatment for Kodamaea alishanica

XML Treatment for Kodamaea arcana

XML Treatment for Kodamaea derodonti

XML Treatment for Kodamaea fukazawae

XML Treatment for Kodamaea fungicola

XML Treatment for Kodamaea hsintzibuensis

XML Treatment for Kodamaea kaohsiungensis

XML Treatment for Kodamaea leandrae

XML Treatment for Kodamaea lidongshanica

XML Treatment for Kodamaea loeiensis

XML Treatment for Kodamaea mesenterica

XML Treatment for Kodamaea plutei

XML Treatment for Kodamaea restingae

XML Treatment for Kodamaea sagamina

XML Treatment for Kodamaea smagusa

XML Treatment for Kodamaea suecica
